# Intravitreal Dexamethasone Implant (IDI) Alone and Combined with Navigated 577 nm Subthreshold Micropulse Laser (SML) for Diabetic Macular Oedema

**DOI:** 10.3390/jcm11175200

**Published:** 2022-09-02

**Authors:** Lisa Toto, Rossella D’Aloisio, Alberto Quarta, Daniele Libertini, Giada D’Onofrio, Chiara De Nicola, Anna Romano, Rodolfo Mastropasqua

**Affiliations:** Department of Medicine and Science of Ageing, Ophthalmology Clinic, University G. D’Annunzio Chieti-Pescara, Via Dei Vestini 31, 66100 Chieti, Italy

**Keywords:** diabetic macular oedema, navigated subthreshold micropulse laser, intravitreal dexamethasone implant

## Abstract

Background: The anatomical and functional changes after intravitreal dexamethasone implant (IDI) alone and combined with navigated subthreshold micropulse laser (NSML) in diabetic macular oedema (DMO) were compared. Methods: Patients with a clinically confirmed diagnosis of non-proliferative diabetic retinopathy (NPDR) and DMO were enrolled in this prospective study and were randomly assigned to two different treatment groups: thirty patients were treated with IDI (IDI group), and the other 30 patients received IDI combined with NSML treatment (combined IDI/NSML group). All patients during a 6-month follow-up underwent best corrected visual acuity (BCVA) evaluation and spectral domain optical coherence tomography (SD OCT). The main outcome measures were: BCVA, central macular thickness (CMT); (3) choroidal vascularity index (CVI), subfoveal choroidal thickness (SCHT); and time to retreatment between IDI at baseline and the second implant in both groups. Results: BCVA, CMT, and SCHT significantly decreased starting from the 1-month follow-up and CVI from 3 months in both groups. The between-group differences were significantly different from 1-month follow-up for BCVA, from 5-month follow-up for CMT and SCHT, and from 4-month follow-up for CVI. The Needed to Treat analysis indicated that six patients would have to be treated with SML after IDI in order for just one person to receive a benefit. Conclusions: the combined treatment showed good anatomical and functional outcomes for the treatment of DMO. In addition, IDI/SML seems to reduce injection frequency over time, improving patients’ quality of life and reducing the socio-economic burden.

## 1. Introduction

Diabetes mellitus (DM) is a metabolic disorder frequently complicated by diabetic retinopathy (DR) and diabetic macular oedema (DMO) [1]. The prevalence of DMO is 0–3% in newly diagnosed diabetic patients, and by 10 years, it occurs in 14 to 25% of DR patients [2,3,4,5,6]. DMO, which can occur at any stage of DR, is one of the leading causes of legal blindness in the working-age population and is the earliest and most common cause of visual loss in patients with DR [5].

Vascular, neurodegenerative, and inflammatory components have been implicated as causes of DMO [7,8,9,10]. In the past, conventional laser photocoagulation demonstrated its efficacy in the prevention of vision loss but did not always consistently improve visual acuity in DR patients with DMO [11].

Currently, intravitreal treatment, either with anti-vascular endothelial growth factor (VEGF) or steroids agents, have become among the most used and effective therapy for DMO condition, with anti-VEGF being the first-line therapy, and intravitreal steroids, particularly intravitreal dexamethasone implant (IDI), being a second line therapy in DMO refractory to anti-VEGF treatments [12].

Recently, Gaweky [13] reported the results of combined treatment of IDI with navigated subthreshold pulse laser (SML) that reduced the number of intravitreal injections in DMO. 

Although the exact mechanism of SML action is not fully understood, it was hypothesized that it stimulates the RPE to repair the inner blood–retinal barrier, probably inducing a modification of the gene expression initiated by the wound healing response with up- and down-regulation of different factors, such as pigment epithelium-derived factor (PEDF), VEGF inhibitors, VEGF inducers, and permeability factors with the final effect of restoring the pathologic imbalance [14,15].

Recent studies reported changes in Muller cell activity biomarkers, reduction in VEGF levels, and impaired expression of cytokines in the aqueous humor of patients with DMO after treatment with SML [16].

There are numerous options for the laser wavelength for SML therapy. Diode lasers that emit at a wavelength of 810 nm are characterized by deep penetration into the choroid, reaching mainly the deeper layers and relatively sparing the neural retina. Yellow lasers that emit at a 577 nm wavelength have the advantage of being minimally absorbed by the xanthophyll, so performing a treatment near the fovea is relatively safe [14].

Recently, non-contact navigated devices improved the efficacy and safety of SML. Navigated laser therapy uses an eye-tracking laser delivery system with the possibility of overlaying retinal images onto the real-time fundus image. Registered image overlays allow the surgeon to map and target precise treatment areas while the eye-tracking system compensates for eye movements. In addition, preset grid patterns with equidistant spacing or confluent spots can be delivered semi-automatically to the planned treatment area with precision, thus increasing the accuracy of laser delivery [17].

Choroid has a central role in diabetic retinopathy. The recent term “diabetic choroidopathy” stands for several choroidal changes in diabetic patients described in the literature over the years; the most recent focus has been on choroidal thickness, which is significantly different from healthy patients. There are also uncertainties about the relationship between the progression of the choroidal thickness (CT) and the severity of diabetic retinopathy. Choroid plays an important role in the rupture of the outer retinal–blood barrier in patients with DMO. Considering choroidal parameters, we aimed to analyze how the choroid could be modified through a single or a double treatment, considering, in particular, a standardized parameter such as CVI, which take into account the vessel and the stromal component of the choroid.

The final aim of this study was to compare the efficacy of IDI alone and combined with navigated 577 nm yellow SML for the treatment of DMO, evaluating functional and anatomical changes. 

## 2. Materials and Methods

### 2.1. Study Participants

In this single-center prospective study, 60 consecutive patients with DM and DR complicated by center-involved DMO who were candidates for IDI were enrolled in the retina center of the Ophthalmology Clinic of University G. D’Annunzio of Chieti-Pescara, Italy. They were diagnosed with DR using color fundus photography, fundus fluorescein angiography (FFA), and spectral domain optical coherence tomography (SD-OCT) and were evaluated with a comprehensive ophthalmologic examination.

This study was approved by the Institutional Review Board (IRB) (Department of Medicine and Science of Ageing, University G. D’Annunzio Chieti-Pescara), and adhered to the tenets of the Declaration of Helsinki. Informed consent was obtained from all patients prior to enrollment. 

The inclusion criteria were: (1) age > 18 years old; (2) diagnosis of type 2 DM with non-proliferative moderate DR (NPDR) according to the simplified version of the ETDRS classification complicated by center-involved treatment-naïve DMO; (3) best-corrected visual acuity (BCVA) greater than 1.0 logMAR in the study eye at baseline examination; (4) central macular thickness (CMT) > 300 μm as measured using the SD-OCT at the baseline examination, and (5) HbA1AC < 7%.

The exclusion criteria were: (1) presence of macular scar and/or epiretinal membrane; (2) other retinal diseases that may cause macular oedema; (3) significant media opacity; (4) cataract surgery in the last 6 months; (5) history of ocular hypertension or glaucoma; (6) previous macular laser treatments; (7) panretinal photocoagulation in last 6 months; and (8) signs or history of uncontrolled blood pressure or neurodegenerative diseases.

### 2.2. Study Protocol

All patients were enrolled between April 2021 and October 2021 and underwent a complete ophthalmologic examination, including BCVA evaluation using the Early Treatment Diabetic Retinopathy Study (ETDRS) chart after refraction measurements, Goldmann applanation tonometry, slit-lamp biomicroscopy, and indirect fundus ophthalmoscopy. In addition, multicolor imaging (MCI) and SD-OCT were performed using Spectralis^®^ HRA + OCT (Heidelberg Engineering, Heidelberg, Germany).

Patients were randomly assigned to two different groups: thirty patients were assigned to IDI (IDI group), and the other 30 patients received IDI followed by SML treatment (combined IDI/SML group).

At T0, all enrolled patients underwent IDI that could be administered for a second time, not before 4 months, and CMT > 300 µm at the discretion of the retinal specialist.

At T2, patients in the combined IDI/SML group underwent a one-time procedure of macular laser treatment using Navigated Yellow 577 nm wavelength SML (Navilas^®^ Laser System 577s Prime, OD-OS GmbH, Teltow, Germany).

All examinations were performed at T0 and every month for 6 months (T1–T6) after the intravitreal dexamethasone implant (IDI). Patients were also asked to return for a visit up to 9 months if there was a recurrence of visual acuity impairment.

### 2.3. SD-OCT Analysis

The acquisition protocol for SD OCT included 49 horizontal raster dense linear B-scans centered on the fovea. A horizontal and vertical B-scans centered on the fovea with enhanced depth imaging (EDI) mode were acquired in all patients. 

All acquisitions following the baseline visit were acquired using the follow-up function. 

Central macular thickness was measured using the central 1 mm-diameter circle of the ETDRS thickness map. 

SD-OCT imaging for choroidal evaluation was performed using EDI mode, which can obtain OCT scans with an axial resolution of 7 μm in tissue. For each subject, a horizontal single-line scan involving the fovea was acquired. All the patients were imaged between 9.00 a.m. and 11.00 a.m., preventing the effect of circadian rhythm on choroidal parameters. Low quality images (signal strength < 23) were excluded and repeated [18].

Subfoveal choroidal thickness (SCHT) measured vertically from the outer border of the RPE to the inner border of the sclera was measured using the inbuilt manual caliper on EDI OCT. Measurements were performed by a single expert grader (L.T). 

For the measurement of the choroidal vascular index (CVI), a previously described approach was applied [19]. Briefly, ImageJ version 1.52q (National Institutes of Health, Bethesda, MD; available at http://rsb.info.nih.gov/ij/index.html, accessed on 2 April 2022) was used for image processing. EDI-OCT images were first converted to 8-bit images using the Default setting. Subsequently, Niblack’s auto local threshold tool was performed to allow segmentation of the luminal area (LA) and stromal area (SA). Then, the total choroidal area (TCA) was delineated using a polygonal tool by manual plotting of the choroidal upper border at the RPE and the lower border marked at the choroid–sclera junction. Then the images were converted back to an RGB (red, green, blue) image to allow computation of the size of LA by the color threshold tool. The CVI (%) was thus calculated as the LA divided by the TCA, and values are included between 0 and 1.

Measurements of SCHT were performed by two experienced ophthalmologists (AQ, DL) with a third (LT) when a consensus was not reached.

### 2.4. Procedures

The intravitreal injection technique of IDI was as follows: sterilization and drabbing, irrigation of the conjunctival sac with diluted betadine solution 5%, followed by injection of the implant 4 mm from the limbus for phakic eyes and 3,5 mm for pseudophakic eyes in the inferotemporal quadrant using the provided 30-gauge injector. Topical antibiotic drops were prescribed to all eyes 4 times/day for 10 days. 

As previously reported [13], The Navilas^®^ Laser System 577s Prime, a 577 nm yellow laser system for navigated focal and peripheral laser treatments (OD-OS GmbH, Warthestraße 21, 14513 Teltow, Germany), was used. The micropulse treatment parameters were standardized for all patients, with 100 μm spot size and 100 ms duration with a 5% duty cycle. The power was individualized in every patient after energy titration before treatment in a normal area of the retina outside the vascular arcade. The titration was performed in microsecond mode with a 5% duty cycle starting from 700 mW power with single spots with 50 mW increasing power until the appearance of a barely visible burn on the retina; this was used as the threshold limit. The final laser treatment power was set at 30% of titrated energy and ranged from 210 to 260 mW. Fixed laser parameters of 100 µm, a duty cycle at 5% and a 0.1 s duration were employed in each case. The micropulse laser in a multiple dense spot pattern was delivered to cover the entire extent of macular edema based on OCT images that were imported to the laser device, superimposed, and aligned with the live image by means of an eye-tracking system. The number of treatment spots varied in every patient, with no spacing application of spots. All procedures were performed by the same experienced clinician (L.T.).

### 2.5. Main and Secondary Outcomes

The main outcome measure was the number of patients requiring a second IDI in both groups. Secondary outcomes were:

(1) Central macular thickness, BCVA, SCHT, and CVI changes during follow-up in both groups;
(2) Time to retreatment between first and second IDI in both groups in order to assess the SML impact on retreatment.

### 2.6. Statistical Analysis

Age, CMT, BCVA, CHT, and CVI were treated as continuous variables and presented as mean ± standard deviation (SD). Categorical variables (sex and patients requiring a second injection) were presented as frequencies and percentages. Variance ratio tests were used to compare the variances of the two groups for each variable. Differences between data sets over time were assessed with paired samples *t*-tests. The Kaplan–Meier method was used to analyze all time-to-event distributions for treated groups. The Kaplan–Meier curve depicted the likelihood of being retreated over a 6-month period. Fisher’s exact test was calculated for the number of patients requiring a second injection. The Number Needed to Treat analysis was calculated to understand how many patients would avoid the necessity of a second injection. In the combined group, MedCalc Software Version 19.3.1 (MedCalc Software Ltd., Ostend, Belgium) was used to perform the Number Needed to Treat analysis. All other statistical analyses were performed with IBM SPSS™ software version 25.0.1 (IBM SPSS Inc., Chicago, IL, USA). A value of *p* < 0.05 was considered statistically significant.

## 3. Results

### Characteristics of Enrolled Patients

All patients successfully completed their assigned treatment and were not lost to follow-up. SCHT measurements did not require a third reader.

**Figure 1:**
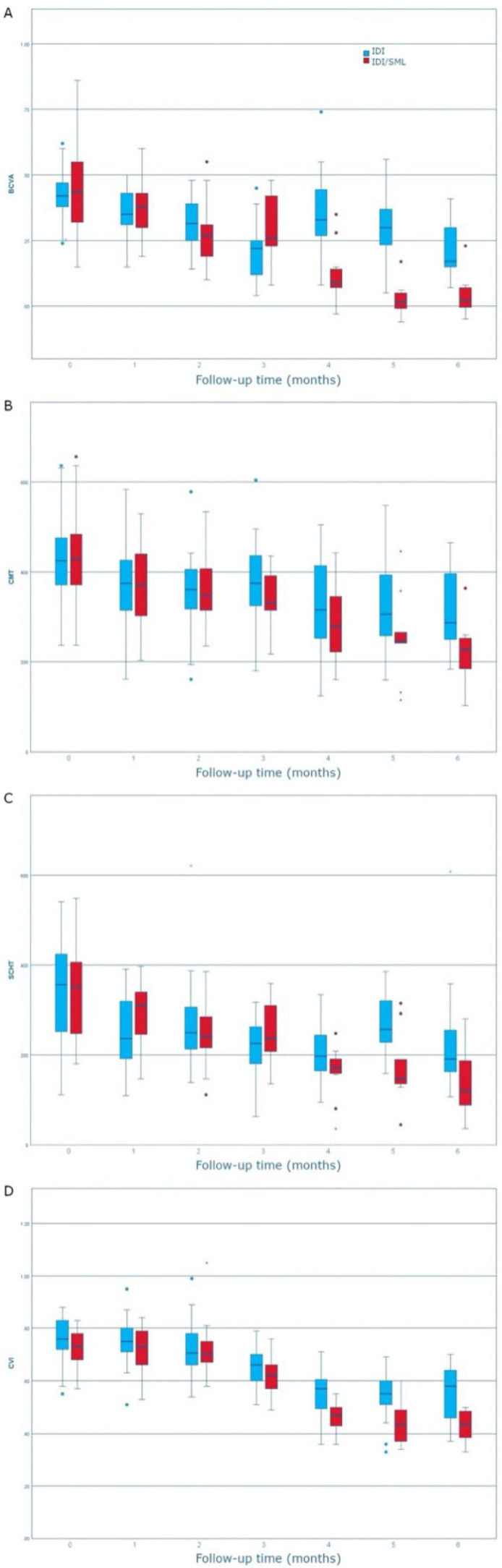
BCVA (**A**), CMT (**B**), SCHT (**C**), and CVI (**D**) variation over time in the two groups. The *p*-values indicate the results of the General Linear Model for Repeated Measures linear trends of time-group effect.

The differences in sex; age; duration of diabetes; and BCVA, CVI, CMT, and SCHT at baseline between the two groups were not statistically significant (Table 1). General Linear Models Repeated Measures analysis of each of the four parameters showed a statistically significant difference in time/group linear effect (Figure 1, Table 2). The trend in the modification of BCVA for the IDI group was variable over time, while the IDI/SML group showed a consistent improvement. The trends for CMT, SCHT, and CVI for both groups were similar, with a greater decrease over time for the latter group. BCVA, CMT, and SCHT significantly decreased starting from the 1-month follow-up and CVI from 3-months in both groups (Figure 1, Table 2). The between-group differences were significantly different from 1-month follow-up for BCVA, from 5-month follow-up for CMT and SCHT, and from 4-month follow-up for CVI.

**Figure 2:**
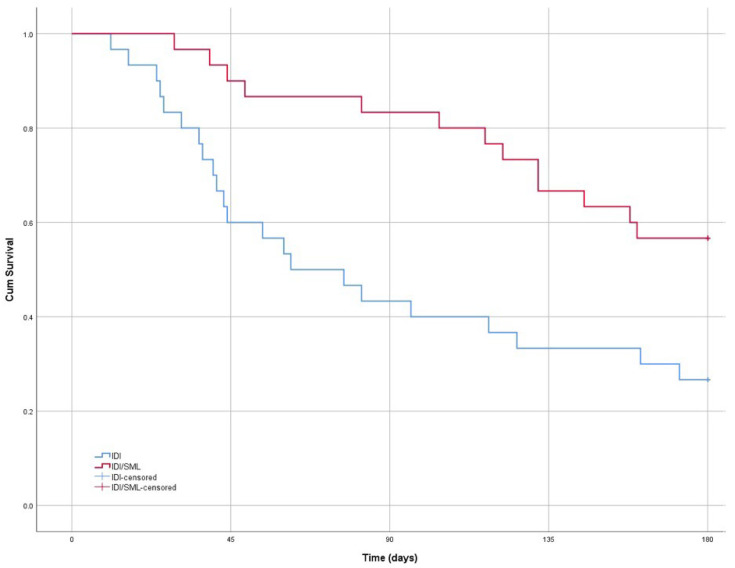
Kaplan–Meier Survival Curve comparison of the two groups showed a statistically significant difference in retreatment.

The number of patients requiring a second injection in the two groups was 22 (73.3%) in the IDI group and 17 (56.7%) in the combined IDI/SML group (Fisher exact test, *p* = 0.279). The Kaplan–Meier Survival Curve comparison of the two groups showed a statistically significant difference (*p* = 0.004, Log Rank (Mantel–Cox), Figure 2). The Needed to Treat analysis indicated that six patients would have to be treated with SML after IDI in order for just one person to receive a benefit (i.e., prevent a second injection during the following 6-month period). The mean time before patients required a second injection was 137.4 days (95% CI: 94.3–180.5) for the combined IDI/SML group and 83.5 (95% CI: 63.3–103.8) days for the IDI group.

## 4. Discussion

This study compared the efficacy of IDI and combined IDI/navigated SML in DMO during a 6-month follow-up study. Both treatments were effective, showing recovery of retinal morphology and improvement of visual function.

Mean BCVA improved significantly, and CMT, SCHT, and CVI reduced significantly over time (*p* < 0.001), showing greater variation in IDI/SML group compared to the IDI group. The between-group differences were significantly different from 1-month follow-up for BCVA, from 5-month follow-up for CMT and SCHT, and from 4-month follow-up for CVI. A lower percentage of patients required a second injection in the combined IDI/SML 17 (56.7%) group compared to the IDI group 22 (73.3%). The mean time before patients required a second injection was 137.4 days (95% CI: 94.3–180.5) for the combined IDI/SML group and 83.5 (95% CI: 63.3–103.8) days for the IDI group.

An important advantage entailed in IDI, compared to intravitreal anti-VEGF injections, is the increased injection-free interval, lasting at least three months [20,21,22].

On the other hand, the recurrence of macular edema, requiring repeated injections over time, is still a considerable issue for the management of DMO patients. Pain experienced by patients due to several surgical procedures may influence patients’ compliance and their decision on whether to continue treatment, eventually leading to a decreased quality of life [23]. Furthermore, the possible development of cataracts necessitating surgery and an intraocular pressure rise may also limit the use of IDI [24].

Recently, SML was introduced as an effective method for the treatment of DMO [3]. 

The efficacy and safety of the subthreshold laser using prevalently a diode source of 810 nm or yellow 577 nm micropulse laser in DMO treatment was demonstrated in several studies in the past decades. In a recent review, Vujosevic et al. [25] found that SML was a new and promising treatment option for DMO, resulting in a safer profile in terms of morphologic and functional parameters.

In a retrospective work conducted with the use of a subthreshold diode micropulse laser to treat DMO, Luttrull et al. [26] demonstrated a beneficial effect on visual acuity and macular oedema resolution. In another study, Nicolò M. et al. [27] reported a statistically significant improvement in BCVA and CRT after one single session of yellow micropulse laser in eyes with DMO.

In our study, we evaluated the efficacy of IDI combined with 577 nm wavelength yellow SML performed 60 days after IDI compared to IDI alone for the treatment of DMO during a 6-month follow-up time. Both treatment options were effective, showing a significant decrease in CMT and a related increase in BCVA. 

In accordance with our results, Elhamid [28], in a prospective study on DMO patients undergoing a one-time procedure micropulse yellow laser one month after an IDI, obtained a significant reduction in CMT.

In a pilot study, Mansouri A. et al. [29] demonstrated that the anatomical severity of DMO may influence the treatment response to SML. They evaluated a total of 63 eyes of 58 patients with diabetic macular edema, dividing them into two groups based on their initial CMT, and reported a lack of response in patients with CMT greater than 400 μm. 

Citirik M. [30] conducted a study on eighty eyes of 40 patients with DMO who were divided into four groups according to their initial CMT values and showed a statistically significant reduction in CMT in the patients with CMT of 300 μm or less.

As reported in previous studies, the efficacy of SML in our patients was probably related to the low retinal thickness obtained 60 days after IDI that in the combined group was 352.04 ± 82.46 µm.

The continuous reduction in CMT in the combined IDI/SML group may be explained by the long-acting effect of both IDI and SML. 

In a prospective study by Luttrull et al. [31], it was shown a reduction in the macular thickness of 59% 3 months after the SML procedure. 

Other studies demonstrated the additional effect of SML combined with intravitreal anti-VEGF injections for the treatment of DMO.

In a prospective, single-center, randomized trial with patients randomly assigned to receive either aflibercept plus micropulse laser or aflibercept monotherapy, Abouhussein M.A. et al. [32] reported that adding 577 nm SML to aflibercept was effective for treatment-naıve DMO and was associated with decreased number of repeated injections. 

In another study, Kanar, H.S. et al. [33] demonstrated that the combination therapy of intravitreal anti-VEGF injection in a pro re nata (PRN) regimen with SML significantly reduced the number of injections required to maintain the resolution of foveal edema in DMO.

As already demonstrated in the literature, we hypothesized that in our study, the greater reduction in CMT in the IDI/SML group compared to the IDI group could be related to the additional delayed effect of SML treatment extending the IDI effect and postponing the need for further treatment.

Increasing the injection-free interval, as obtained in our study, may translate into a reduced socio-economic burden for the national health system as well as a reduced number of outpatient hospital access, improving patients’ quality of life. In a previous study assessing the compliance of patients with DMO treated with ranibizumab in a real-life setting, it was shown that the visual outcomes correlate with the number of intravitreal injections and that a strict monthly follow-up is challenging in real-life [34].

Interestingly a reduction in SCHT and CVI was also found in our sample in both groups, with a greater reduction in the combined group compared to the IDI group.

Altun et al. demonstrated a statistically significant thinning of the mean subfoveal choroidal thickness in the follow-up period after IDI in vitrectomized diabetic eyes with DME, and they hypothesized that this could occur due to the decrease in vasodilator effect of proinflammatory cytokines and mediators [35].

Aksoy et al. also demonstrated a reduction during a 3-month follow-up of subfoveal choroidal thickness measurements in patients with DMO treated with IDI or intravitreal injection of aflibercept [36].

In another study, CVI was found to be reduced after IDI implant in patients with DME during a 3-month follow-up [37].

Dexamethasone surely reduces the number of cytokines involved in this process, and recently Midena et al. [8] demonstrated that sub-threshold micropulse laser treatment probably reduces inflammatory biomarkers in aqueous humor of diabetic patients with macular oedema. 

The greater reduction in SCHT and CVI in the combined IDI/SML group was probably related to an additional effect of SML on the modulation of cytokines and growth factors, reducing choroidal permeability.

This study presents several limitations. First, the sample size was not calculated because prior results with IDI and NSML were not available. Second, the results obtained in this single-center design will have to be confirmed in a multicenter study. Third, the follow-up was short, and a longer follow-up would better clarify the long-term efficacy of SML and the possible additive effects of repeated SML treatments. However, the main advantage of the study is its prospective nature alongside being the first one, to our knowledge, to assess among treatment outcomes the changes in CVI after a combined IDI and SML therapy in DME eyes.

In conclusion, our study demonstrated in a short follow-up period the efficacy of combining SML with IDI for the treatment of DMO as an adjunctive option to make IDI implants last longer and thus delay DMO recurrence. This could translate into a better quality of life for patients reducing the burden of intravitreal treatment.

## Figures and Tables

**Table 1:** Characteristics and anatomical parameters of enrolled patients. All values are expressed as median (1st quartile; 3rd quartile). *p*-value results from the nonparametric test for independent samples.

	IDI/SML	IDI	*p*-Value
Number of eyes	30	30	-
Age, (years)	70.5 ± 11.8	72.8 ± 9.9	0.417
Sex, (M/F)	18/12	14/16	0.438
Diabetes duration, (years)	14.7 ± 8.9	15.5 ± 8.5	0.723
BCVA, (logMAR)	0.46 ± 0.17	0.43 ± 0.09	0.403
CMT, (µm)	430.73 ± 103.39	422.97 ± 95.90	0.766
SCHT, (µm)	342.63 ± 103.56	340.66 ± 111.02	0.944
CVI	0.73 ± 0.07	0.76 ± 0.08	0.096

IDI: intravitreal dexamethasone implant; IDI/SML: intravitreal dexamethasone implant/subthreshold micropulsed laser; BCVA: best corrected visual acuity; CMT: central macular thickness; CVI: Choroidal vascularity index; SCHT: subfoveal choroidal thickness.

**Table 2:** Mean and standard deviation of the percentage variation from baseline for each anatomical and functional parameter. The *p*-values indicate the results for the paired sample *t*-test for each time point versus baseline.

	T1	T2	T3	T4	T5	T6
**IDI**						
BCVA, (logMAR)	12.40 ± 24.99	25.76 ± 16.57	53.29 ± 16.98	−8.97 ± 32.81	7.96 ± 37.28	28.61 ± 27.23
*p*	0.003	<0.001	<0.001	0.016	<0.001	<0.001
CMT, (µm)	7.41 ± 24.73	15.41 ± 16.97	9.73 ± 26.04	-3.28 ± 31.08	5.08 ± 32.80	10.14 ± 29.11
*p*	0.004	<0.001	0.031	<0.001	0.001	<0.001
SCHT, (µm)	17.19 ± 45.27	15.03 ± 34.56	28.73 ± 38.10	19.96 ± 56.64	16.70 ± 42.31	32.58 ± 35.11
*p*	<0.001	<0.001	<0.001	<0.001	0.001	<0.001
CVI	1.09 ± 16.13	4.17 ± 17.88	13.58 ± 15.55	0.70 ± 15.16	9.01 ± 25.61	7.63 ± 30.04
*p*	0.443	0.132	<0.001	<0.001	<0.001	<0.001
**IDI/SML**						
BCVA, (logMAR)	13.44 ± 41.34	37.01 ± 36.20	39.29 ± 25.55	56.70 ± 15.80	77.17 ± 7.61	73.49 ± 7.11
*p*	<0.001	<0.001	<0.001	<0.001	<0.001	<0.001
CMT, (µm)	11.97 ± 24.02	15.79 ± 16.58	22.79 ± 19.15	14.88 ± 17.57	23.78 ± 15.71	29.01 ± 5.38
*p*	0.002	0.001	0.001	<0.001	<0.001	<0.001
SCHT, (µm)	14.86 ± 23.05	27.60 ± 16.05	30.38 ± 8.73	22.43 ± 18.99	22.69 ± 7.10	27.89 ± 3.73
*p*	0.002	<0.001	<0.001	<0.001	<0.001	<0.001
CVI	0.87 ± 13.79	−0.70 ± 26.18	14.12 ± 11.46	−4.96 ± 14.26	−2.57 ± 19.45	−1.28 ± 11.86
*p*	0.566	0.789	0.001	<0.001	<0.001	<0.001

BCVA: best corrected visual acuity; CMT: central macular thickness; CVI: Choroidal vascularity index; SCHT: subfoveal choroidal thickness.

## Data Availability

Not applicable.
